# Using Fenestrated Stent to Increase the Flow of Extracorporeal Membrane Oxygenation of Superior Vena Cava Compression Syndrome

**DOI:** 10.7759/cureus.46008

**Published:** 2023-09-26

**Authors:** Nafiye Busra Celik, Ishaq J Wadiwala, Mustafa Sadek, Ramez Ibrahim, Mohammad Alomari, Emad Alamouti-fard, Lekhya Raavi, Md Walid Akram Hussain, Samuel Jacob

**Affiliations:** 1 Department of Cardiothoracic Surgery, Mayo Clinic, Jacksonville, USA; 2 Department of Cardiothoracic Surgery, Heart and Lung Transplant National Recovery Program, Jacksonville, USA

**Keywords:** endovascular stent therapy, superior vena cava (svc) obstruction, increase flow of ecmo, fenestrated stent, ecmo outflow due to svcs, mediastinal tumors, va-ecmo, superior vena cava (svc) syndrome

## Abstract

Superior vena cava syndrome (SVCS) is an obstruction of the venous return through the superior vena cava (SVC) or any other significant branches. The obstruction may be external, like thoracic mass compressing the SVC, or internal, like thrombosis or tumor, which directly invades the SVC. Patients experiencing a medical emergency after being initially stabilized require treatment for SVCS, including endovenous recanalization and the implantation of an SVC stent to reduce the risk of abrupt respiratory arrest and death.

A 54-year-old female presented from the university medical center with weight loss and solid food dysphagia for three months. Chest-CT scan showed a mediastinal mass of 10 x 9 x 8 cm. A transbronchial biopsy was attempted. The patient was arrested during the bronchoscopy lab procedure. Cardiopulmonary resuscitation (CPR) was initiated, and venoarterial-extracorporeal membrane oxygenation (VA-ECMO) was done through the right femoral artery cannula size 15 Fr due to the narrowing of the artery and the left femoral vein cannula size 23 Fr. During the night shift, the ECMO flow was hard to maintain with fluids, which was realized with the ECMO outflow volume issue. The next day, in the hybrid operating room, a fenestrated SVC stent was placed in the SVC, brachiocephalic, and internal jugular veins. The patient's hemodynamics improved post-stenting, especially ECMO outflow.

This case illustrates that stenting in SVCS is a valid therapeutic option to increase the ECMO flow in this patient group.

## Introduction

Superior vena cava syndrome (SVCS) is a blockage of the superior vena cava (SVC) caused by external compression, neoplastic invasion, or internal obstruction. Most blood that leaves the head, neck, upper extremities, and upper thorax travels to the heart through the SVC. Edema and plethora of the face, neck, chest, and upper extremities; dilated veins in the same regions; wheezing and coughing; headache; and hoarseness are typical signs and symptoms of SVCS [1]. Endovascular therapy, which includes stenting, percutaneous transluminal angioplasty, and intravascular thrombolysis, is one of the potential treatments for SVCS [2]. This case report describes a scenario in which a fenestrated SVC stent was used to improve the decreased flow in a venoarterial (VA)-extracorporeal membrane oxygenation (ECMO) outflow due to SVCS.

This article was previously presented as a meeting abstract at the 2023 American Society for Artificial Internal Organs (ASAIO) 68th Annual Conference on June 14, 2023.

## Case presentation

**Figure 1:**
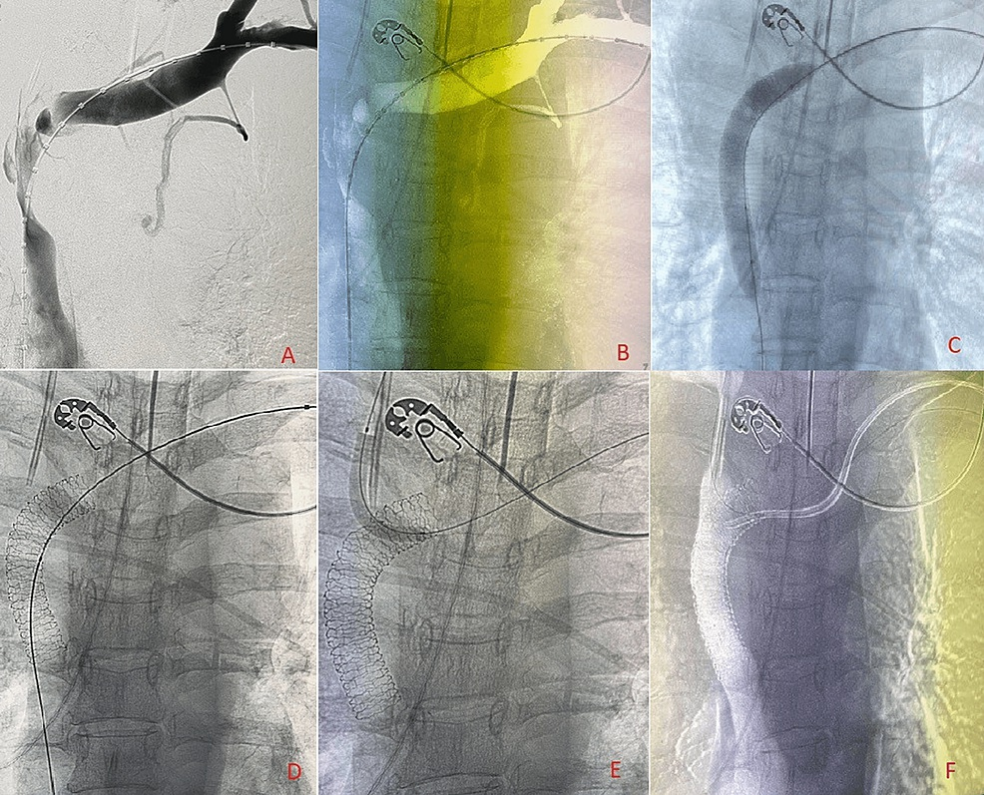
SVC stenosis and deployed stents under fluoroscopy (A) Fluoroscopy showing SVC stenosis. (B) Redacted view of SVC fluoroscopy. (C) Balloon angioplasty of SVC stenosis under fluoroscopy. (D) Fenestrated stent placement in the SVC under fluoroscopy. (E) Stent deployed internal jugular. (F) Post-stenting image showing deployed brachiocephalic and internal jugular veins through fenestrations of the primary stent. SVC: Superior vena cava.

A 54-year-old female was referred to our hospital with CT imaging with suspicion of a new mediastinal mass. She had a recent history of significant weight loss and solid food dysphagia, dry cough, and occasional dyspnea, all of which did not improve with conservative management over the past three months. After persistent symptoms, further CT chest detected a 10 x 9 x 8 cm mediastinal mass compressing the trachea, bilateral mainstem bronchus, and SVC. Transbronchial biopsy was decided to proceed as the next course of treatment. CT-guided biopsy showed a large lymphoma with multiple sites. However, the patient had a cardiac arrest during the procedure in the bronchoscopy lab. The ECMO team was contacted for cannulation after starting CPR. Due to the arterial calcification, VA-ECMO cannulation was done using a downsize from 17 Fr to 15 Fr for the right femoral artery and a left femoral vein cannula size 23 Fr. After the return of spontaneous circulation (ROSC) was restored, the patient was transferred to the ICU. It was challenging to sustain the ECMO flow with fluids during the night shift, and the small artery cannula was suspected as the reason, but it was later determined that the problem was with volume. A bedside bronchoscopy showed left mainstem bronchus compression and a patent right mainstem bronchus with minimum secretions. The next day, the patient was taken into a hybrid operating room, and the initial fluoroscopy showed SVC stenosis (Figure 1, Panels A and B). Balloon angioplasty was performed on the stenotic segment (Figure 1, Panel C). A primary fenestrated stent was then deployed in the SVC (Figure 1, Panel D), and two additional stents were deployed through the fenestrations into the internal jugular and brachiocephalic veins (Figure 1, Panels E and F). The final fluoroscopy shows patent SVC with deployed stents in the problematic veins (Figure 1, Panel F), and this was reflected in the hemodynamics, including ECMO outflow, which considerably improved immediately after the procedure. The patient was transferred to the ICU for hemodynamic monitoring immediately after the procedure.

## Discussion

The SVC is a vessel that drains blood from the upper body. It is a low-pressure system with a highly compressible thin wall. Extrinsic compression is the main reason for SVC syndrome. Most often, mediastinal malignancies, such as bronchogenic carcinoma and non-Hodgkin's lymphoma, are responsible for it. Around 20% of cases have non-malignant origins. It contains pericarditis, benign tumors, aortic aneurysms, thrombosis, mediastinal fibrosis, and infections like TB [3].

Endovascular repair (EVR) is increasingly preferred as the first line of treatment for benign SVC conditions, and it has become more common for all vascular issues. A review from Rizvi et al. [4] showed that their practice has also changed significantly, with most patients attempting EVR as their primary treatment. An endovascular intervention had either failed or been unsuccessful in eight of the 13 patients who underwent surgery in the previous five years.

On the other hand, open surgery has limited applications, including benign diseases, unfavorable anatomy for endovascular treatment, and failed endovascular therapy [4]. Open surgery may still be the best course of action in some circumstances, though, depending on the patient and their prognosis. However, the most prevalent cause of SVC excision and repair is malignant invasion, and these are indicated for prolonged survival [5].

There is no consensus regarding the selection of a stent; however, the choice of the stent is often determined by the etiology and severity of the stenosis. The most common stents are Palmaz (Cordis, Hialeah, Florida), Wallstent (Boston Scientific, Marlborough, Massachusetts), and Gianturco Z-stent (Cook, Bloomington, Indiana) are used, with Wallstent being preferred since it is self-expanding and non-compressible. Stents having a diameter of 18-22 mm are currently suggested [6,7]. Briefly, the Palmaz design balloon-expandable intraluminal stent (BEIS) and self-expanding Gianturco Z-stents are made of stainless steel wire that has been twisted into an open cylinder using a sequence of "Z" curves. Studies have shown that 80%-100% of patients who have the Gianturco Z-stent placed in their SVC get instant symptom alleviation [8,9]. In this case, the patient's condition was improved by the placement of the internal jugular and brachiocephalic veins stents through a fenestrated main stent in the SVC. Additionally, the ECMO outflow improved immediately compared to the initial sluggish flow. The use of extracorporeal membrane oxygenation (ECMO) for the support of patients who do not respond to traditional cardiopulmonary resuscitation is what is known as extracorporeal cardiopulmonary resuscitation (ECPR). Regarding this, Mosca et al. showed that ECPR increased refractory patient survival rates related to those who had traditional resuscitation before ECPR [10]. ECMO should be addressed when medical therapy is ineffective for individuals with severe, acute, reversible respiratory failure. Increasing systemic oxygenation and CO_2_ elimination (ventilation) and reducing the need for harmful mechanical ventilation are two aspects of the physiologic justification for using ECMO [11].

## Conclusions

Endovascular therapy can effectively relieve the obstructive symptoms of SVCS, particularly in emergencies, by employing angioplasty and stenting while providing prompt relief from sluggish ECMO outflow, as encountered in our case above.
